# Unexpected Conduction Blocks in *SORD* ‐Related Distal Motor Neuropathy: A Case Report

**DOI:** 10.1002/ccr3.73401

**Published:** 2026-09-01

**Authors:** Amiela Angeli Bucur, Gabriele Rodolico, Alessandra Tessa, Filippo Maria Santorelli, Martina Sperti, Deborah Leccese, Mario Maturo, Sabrina Matà

**Affiliations:** ^1^ Department of Neuroscience, Psychology, Drug Research and Child Health (NEUROFARBA) University of Florence Florence Italy; ^2^ Molecular Medicine for Neurodegenerative and Neuromuscular Diseases Unit IRCCS Stella Maris Foundation Pisa Italy

**Keywords:** conduction block, distal motor neuropathy, distal muscle atrophy, electromyography, peripheral neuropathy, SORD

## Abstract

Distal weakness requires an extensive differential diagnosis. In this case, conduction blocks indicated multifocal motor neuropathy, but immunotherapy proved ineffective. Ultimately, genetic testing revealed a sorbitol dehydrogenase mutation, highlighting the importance of combining electrophysiological findings with molecular studies in cases where the diagnosis remains unclear.

## 1 Introduction

Approaching a patient with distal lower limb muscle weakness can be particularly challenging due to the wide range of conditions that can impair distal muscle strength. Acquired conditions include autoimmune neuropathies, such as pure motor chronic inflammatory demyelinating polyneuropathy (CIDP) and multifocal motor neuropathy (MMN), and degenerative diseases, including amyotrophic lateral sclerosis (ALS). Inherited disorders, such as distal muscular dystrophy, distal hereditary motor neuropathies (dHMN), and hereditary ALS, must also be considered. The present case illustrates the difficulty in making a definite diagnosis in such cases.

## 2 Case History/Examination

**FIGURE 1:**
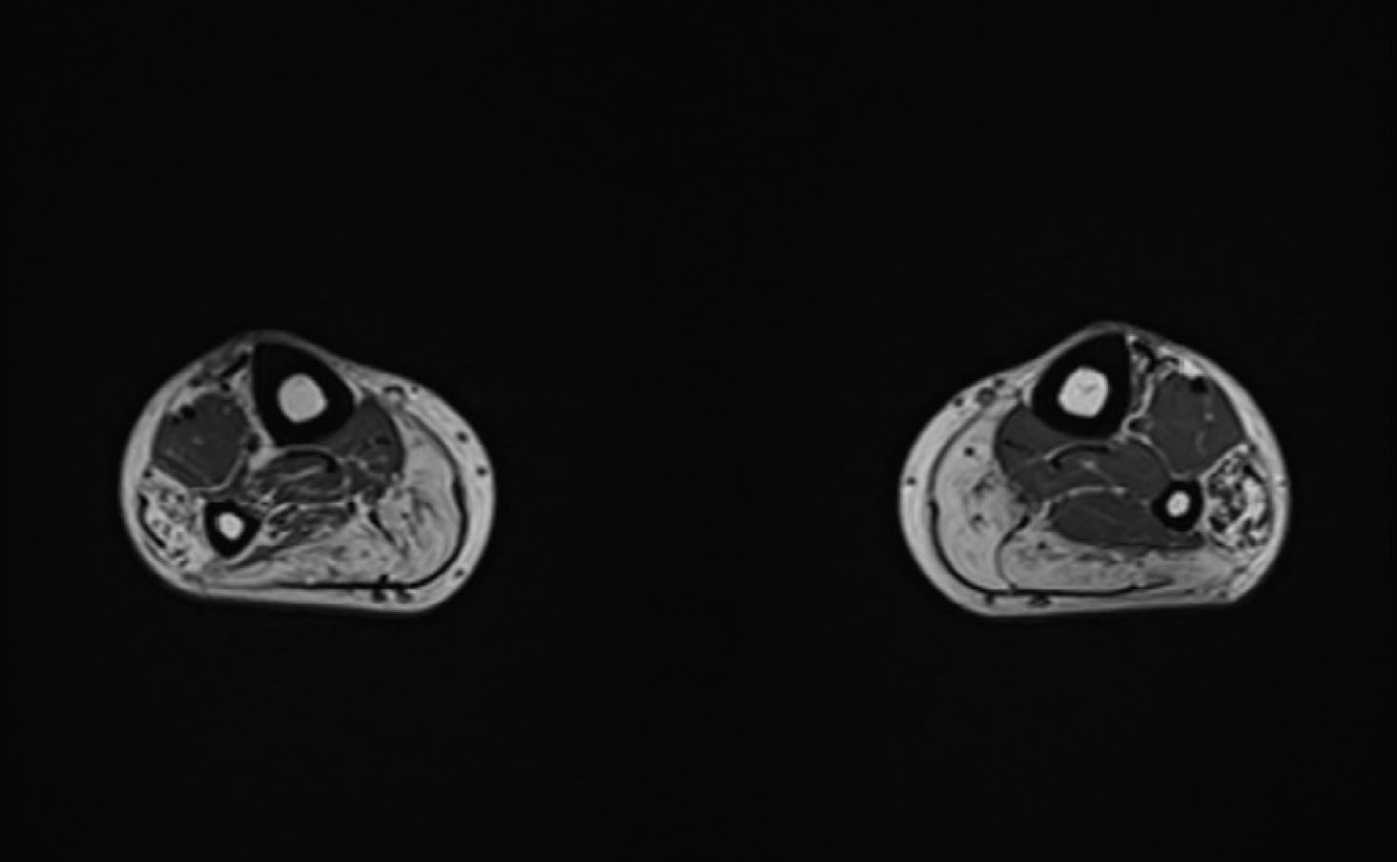
Gross fatty atrophy of bilateral gastrocnemius and peroneus longus muscles with near complete fatty replacement. Moderate fatty atrophy of the soleus muscles is seen (right>left).

A 36‐year‐old male presented in March 2021 with a 6‐year history of weakness in the lower limbs that had slowly progressed up to the distal upper limb. He reported no family history of neurological disorders. The neurological examination showed distal muscular atrophy in the lower limbs, with steppage gait. Mild weakness of the intrinsic hand muscles was also observed. Tendon reflexes were absent in the lower limbs and normal in the upper limbs. Nerve conduction study (NCS) showed a 51.2% drop in the amplitude of the compound motor action potential (CMAP) of the left ulnar nerve at the forearm and a 63.5% drop in the right ulnar nerve, without a significant increase in the CMAP duration or a decrease in the motor conduction velocity, suggesting a definite motor conduction block (Figure 1). The presence of a Martin–Gruber anastomosis was excluded bilaterally. Additionally, a bilateral reduction in CMAP amplitude was registered in the soleus muscle bilaterally. Sensory NCS gave normal results. Electromyography (EMG) showed chronic denervation and reinnervation in the lower limbs involving proximal and distal group muscles, and spontaneous activity in the distal left upper limb extremity, suggesting ongoing denervation. Motor evoked potentials (MEPs) were normal. Laboratory tests, including thyroid function, protein electrophoresis, glucose, and autoantibody screening (including anti‐GM1 antibody) were within normal ranges. A lumbar puncture showed a mild increase in the albumin ratio to 10,6 (normal range < 7). Brain and spinal MRI studies were unremarkable.

## 3 Differential Diagnosis

Following the suspicion of MMN, the patient was given a six‐month treatment with intravenous immunoglobulin. At follow‐up, the motor deficit and NCS findings were unchanged. A comprehensive genetic testing panel for inherited neuropathies was then performed, which showed a heterozygous mutation (c.1010 + 4_1010 + 7 dupAGTT) in SETX, leading to the suspicion of hereditary ALS. However, the same mutation was also detected in the patient's mother, whose neurological examination was unremarkable. A muscle MRI was performed, which showed marked muscle fatty degeneration, predominantly in the posterior calf muscles and, to a lesser extent, in the vastus lateralis and in the posterior thigh muscles. Genetic testing identified a homozygous variant, leading to the diagnosis of *SORD*‐related dHMN.

## 4 Results/Conclusion

The patient presented with distal lower limb weakness and conduction blocks on nerve conduction studies (NCS), as well as EMG signs of chronic denervation (Table 1). This initially suggested multifocal motor neuropathy. However, immunoglobulin therapy was ineffective, and the SETX variant was ruled out as the cause. Final genetic testing identified a homozygous SORD mutation, confirming a diagnosis of SORD‐related distal hereditary motor neuropathy. At follow‐up, the patient remained clinically stable.

**TABLE 1:** Conduction block on nerve conduction studies (motor conduction velocity).

Nerve/positions	Latency ms	Amp. 2–4 mV	Dur. 1–3 ms	Area 1–3 mVms	Dist. cm	Vel. m/s
Right Ulnar‐FDI						
Wrist	3,80	14,8	5,30	28,5		
B. Elbow	7,65	5,4	5,80	12,6	25	64,9
A. Elbow	9,00	4,6	6,10	10,5	7,5	55,6
Axilla	11,65	3,9	5,70	9,6		
Left Ulnar‐FDI						
Wrist	3,75	12,9	6,20	33,8		
B. Elbow	7,80	6,3	7,00	17,6	25	61,7
A. Elbow	9,30	4,9	8,20	14,6	9	60,0
Axilla	10,90	7,5	7,30	19,5		

## 5 Discussion

This case illustrates the challenges of diagnosing an adult patient with distal muscle weakness due to the variety and clinical overlap of associated disease presentations. MMN is an asymmetric, purely motor neuropathy associated with motor conduction blocks; anti‐GM1 antibodies are detected in 40% of cases [1]. In view of the partial motor conduction block at NCS, MMN was first considered as a possible diagnosis for our patient. However, after 6 repeated courses of IVIG there was no significant clinical benefit, although some subjective improvement was reported. This observation, along with the atypical distribution of the motor deficit and the clinical history of a slowly progressive, mainly distal and symmetric disorder, suggested the alternative diagnosis of dHMN. The expanded NGS panel ultimately identified the common variant in SORD. Recently, biallelic mutations in SORD have been identified as a common genetic cause in autosomal recessive CMT patients [2]. SORD is involved in the polyol pathway that converts sorbitol to fructose. Pathogenic variants in SORD determine decreased levels of the enzyme, which consequently causes sorbitol accumulation. It has been shown that increased intracellular sorbitol can lead to selective degeneration of peripheral axons [3] although the neuropathic mechanism remains to be determined. Sorbitol accumulation is historically associated with diabetic neuropathy. It has been shown that, in a diabetic mouse model, pathological nerve changes occur with the accumulation of sorbitol [4], and that these changes may be prevented by the inhibition of aldose reductase. It is interesting that diabetic neuropathy may sometimes have NCS changes consistent with demyelination, including temporal dispersion and partial conduction block [5]. This finding may explain the partial conduction block observed in our patient, a finding already seen in other SORD‐related cases [6].

## Author Contributions

**Amiela Angeli Bucur:** conceptualization, data curation, investigation, writing – original draft. **Gabriele Rodolico:** conceptualization. **Alessandra Tessa:** investigation. **Filippo Maria Santorelli:** conceptualization, investigation, writing – review and editing. **Martina Sperti:** conceptualization. **Deborah Leccese:** conceptualization. **Mario Maturo:** conceptualization. **Sabrina Matà:** conceptualization, investigation, supervision, writing – original draft, writing – review and editing.

## Funding

The authors have nothing to report.

## Ethics Statement

We confirm that we have read the Journal's position on issues involved in ethical publication and affirm that this report is consistent with those guidelines.

## Consent

Written informed consent was obtained from the patient to publish this report in accordance with the journal's patient consent policy.

## Conflicts of Interest

The authors declare no conflicts of interest.

## Data Availability

The data that support the findings of this study are available on request from the corresponding author. The data are not publicly available due to privacy or ethical restrictions.
